# Diagnostic criteria for MOGAD

**DOI:** 10.1007/s00415-024-12405-1

**Published:** 2024-05-17

**Authors:** Sophie Voase, Neil P. Robertson

**Affiliations:** 1 Department of Neurology, University Hospital of Wales, Heath Park, Cardiff, CF14 4XN UK; 2 Division of Psychological Medicine and Clinical Neuroscience, Department of Neurology, Cardiff University, University Hospital of Wales, Heath Park, Cardiff, CF14 4XN UK

## Introduction

Myelin oligodendrocyte glycoprotein antibody-associated disease (MOGAD) is a rare antibody-mediated inflammatory demyelinating disorder of the central nervous system with a broad clinical spectrum. Distinct from multiple sclerosis (MS) and aquaporin-4-seropositive neuromyelitis optica spectrum disorder (AQP4-NMOSD), MOGAD can follow a monophasic or relapsing disease course and is characterised by the presence of antibodies against MOG. Until now, there had been no international consensus on diagnostic criteria for MOGAD and in this month’s journal club, we explore three recent papers on this topic.

The first paper outlines the diagnostic criteria laid out by an international expert panel. The second and third papers report the retrospective systematic application of the 2023 MOGAD diagnostic criteria to regional clinic-based cohorts.

## Diagnosis of myelin oligodendrocyte glycoprotein antibody-associated disease: International MOGAD Panel proposed criteria

A panel composed of international experts from paediatric and adult neurology, immunology and research disciplines examined current evidence and proposed a consensus diagnostic criteria for MOGAD. The 378 papers examined included those identified by a MEDLINE search between 2010 and 2021, articles put forward by individual members of the working groups and older, pivotal studies identified through a review of the papers from the original MEDLINE search. To diagnose MOGAD, the paper lays out a three-step approach involving:

1. Identification of a core clinical demyelinating event
2. A positive MOG-IgG test (if the test is not clear positive, then other supporting clinical/radiological features must be met to proceed)
3. Exclusion of other diagnoses e.g. MS and AQP4-NMOSD

The paper is structured to allow for an examination of the evidence which is used to form the recommendations of the panel and the diagnostic criteria. The paper reviews the most common clinical phenotypes found in patients with MOGAD; optic neuritis, transverse myelitis, and brain/brainstem involvement, together with the clinical features associated with each presentation. The evidence presented here forms the basis for the supporting criteria which must be met if the patient does not have a clear positive result by serum MOG-IgG testing.

After a review of serology testing, the paper recommends that testing for suspected MOGAD should be carried out through serum testing using live cell-based assays using full-length human MOG-IgG. The authors proposed clear criteria for clear positive and low positive fixed and live cell-based assay results. The authors recommended testing at the time of the incident event before initiation of acute management. If an initial test was negative and was performed after acute management had commenced, repeat testing is recommended after an interval of at least 3 months.

The panel also recommend that patient selection needed to be more focused to improve the positive predictive value of testing, recommending that tests should be carried out in patients who have clinical and/or radiological features in keeping with MOGAD rather than all patients with demyelinating disease. They also noted that almost 50% of paediatric patients with optic neuritis/ADEM were MOG-IgG positive compared to 5% of adults with optic neuritis. They suggest that only testing patients with certain features, including severe optic disc oedema or longitudinally extensive optic nerve lesions, amongst others, would increase the diagnostic yield of MOG tests and recommend that MOG testing should not be requested as part of routine screening for patients suspected of having MS due to a 0.3–2.5% false positive rate.

### Comment:

This paper forms the first international, consensus diagnostic criteria for MOGAD and requires validation in existing cohorts. The panel lays out clear criteria for the identification of patients with MOGAD which will allow for better longitudinal evaluation of MOGAD. If all future cohorts include patients that are classified by the same criteria, the consistency and validity of comparisons between different groups will improve. As MOGAD remains a relatively rare disease, having consensus diagnostic criteria is also important to allow for collaboration between groups and is an important step towards clinical trials to optimise clinical management and explore new therapeutic options.

Banwell B et al. The Lancet 2023; 22(3): 268–282. 10.1016/S1474-4422(22)00431-8. Epub 2023 Jan 24. PMID: 36,706,773.

## Validation of the international MOGAD panel proposed criteria

Validation of new diagnostic criteria is vital to prove utility in real-world settings. The new MOGAD criteria were retrospectively applied to 100 patients who presented to a single centre between 2007 and 2023. The criteria were applied to patients who had MOG-IgG testing at the time of the first attack (defined as MOG-IgG testing within 4 months of disease onset) or during the follow up period. The population consisted mainly of adults (84%), 66% were female and all were of Asian ethnicity. The patients were followed up for a median of 23.9 months, with 45% of patients following a monophasic course. 71 patients received maintenance immunotherapies, of whom 66% followed a relapsing disease course.

The cohort consisted of 36 patients with a clear positive MOG-IgG, 27 low positive and 37 who had a positive MOG-IgG without a titre. 61 patients had MOG-IgG testing at the time of the first attack. 84% of patients met the criteria at the first attack including 100% of the ‘clear positive’ group; 93% of patients met the criteria over the entire disease course. The 7 patients who did not meet the diagnostic criteria followed a monophasic course; 4 presented with a short segment myelitis and 3 had unilateral optic neuritis. The most common presenting feature was optic neuritis (43%) followed by transverse myelitis (28%). The group found that 4 patients who initially tested negative for MOG-IgG after receiving steroids, tested positive later (2 tested in relapse, 2 tested during follow-up).

### Comment:

This study validated the diagnostic criteria and provided useful evidence related to the timing of MOG-IgG testing. The authors noted that not all patients met the criteria at the first presentation. However, 93% of their patients met criteria during follow-up. They offer further evidence for the influence of steroid use and timing of sample on serostatus, providing more evidence that tests should be performed before initiation of steroids and early on presentation. The findings are limited by the retrospective design and lack of a control cohort. Another limitation is that the cohort is from a single centre and is made up of predominantly adult patients of a single ethnicity. Further work needs to be done to validate these criteria in other representative populations.

Kim KH et al. Mult Scler 2023;29(13): 1680–1683. 10.1177/13524585231198754. Epub 2023 Sep 20. PMID: 37,728,329.

## Timing of MOG-IgG testing is key to 2023 MOGAD diagnostic criteria

In this study, the new MOGAD diagnostic criteria were retrospectively applied to 408 patients referred to the NMOSD specialised service between 2012 and 2023 with an atypical demyelinating syndrome. The pre-existing diagnoses of this group consisted of MOGAD (127), AQP4-IgG NMOSD (125), seronegative NMOSD (33), MS (10) and other diagnoses (113). Of patients with a pre-existing MOGAD diagnosis, 97% met the new 2023 diagnostic criteria. Of those who did not meet the criteria, 3 did not meet the supportive features and in 1 patient, an alternative diagnosis could not be excluded due to an MS-MOGAD overlap. In the MOGAD group, 103 patients had clear positive serum MOG-IgG tests, 1 patient was serum negative but CSF MOG-IgG positive and 23 had low positive serum MOG-IgG tests. None of the patients with a non-MOGAD diagnosis before validation fulfilled diagnostic criteria which suggested a sensitivity of 97% and specificity of 100%.

Timing of MOG-IgG tests has been shown previously to be important in MOG-IgG serostatus and this paper provided further evidence to that effect. The low-positive results were associated with a significantly longer time from disease onset to sampling (p < 0.001). Only 33/127 of the MOGAD group were tested within 30 days of their onset of clinical syndrome. Of this group, 18 had a repeat test 6–12 months later, with only 4 remaining clear positive at 12 months. In a Kaplan–Meier analysis of patients who underwent testing within 6 months of clinical onset, approximately 25% became low positive by 6 months and approximately 50% became seronegative by 1 year.

### Comment:

This retrospective, single-centre cohort study provides further evidence to validate the utility of the new diagnostic criteria. The retrospective design introduced referral bias so future work should look to a multi-centre, prospective study design. The population consisted of primarily adult patients, but there is limited demographic information included. Since MOGAD has a higher incidence in children than adults the cohort may not be considered representative of the wider population with MOGAD. The study was also unable to compare the performance of the diagnostic criteria in an adult population compared to a paediatric population. Future studies should look at validating the criteria in both paediatric and adult cohorts.

One of the strengths of this paper was the use of an atypical demyelination cohort which demonstrated the specificity of the MOGAD diagnostic criteria. The criteria performed well, with 97% of cases considered to be MOGAD being identified and with no false positives. This study also highlighted the importance of sending MOG-IgG tests early in the disease course if there is a clinical suspicion due to the rate of seroconversion to low positive/negative. This highlights the importance of early expert assessment.

Forcadela M et al. Neurol Neuroimmunol Neuroinflamm 2023;11(1): e200183. 10.1212/NXI.0000000000200183. Print 2024 Jan. PMID: 37,977,848

## Conclusion

This month’s journal club explored the new diagnostic criteria proposed by an international panel and two papers validating these criteria amongst their own cohorts. They found that the criteria are highly sensitive and specific suggesting that they will be useful in the diagnosis of MOAGD going forward. They also provide further evidence on the influence of timing on MOG-IgG serostatus. Currently, there is no unified consensus on the treatment of MOGAD. Future trials should utilise these criteria as a basis for ensuring their cohorts are consistent to allow for multi-centre clinical trials.

## Data Availability

Not relevent for this article.

